# Scientometric overview of nursing research on pain management

**DOI:** 10.1590/1518-8345.2581.3051

**Published:** 2018-09-03

**Authors:** Hale Turhan Damar, Ozlem Bilik, Guzin Ozdagoglu, Aşkın Ozdagoglu, Muhammet Damar

**Affiliations:** 1 MSc, Researcher, Dokuz Eylul University, Faculty of Nursing, İzmir, Turkey.; 2 PhD, Professor, Dokuz Eylul University, Faculty of Nursing, İzmir, Turkey.; 3 PhD, Associate Professor, Dokuz Eylul University, Faculty of Business, Izmir, Turkey.; 4 MSc, Researcher, Dokuz Eylul University, Faculty of Business, Izmir, Turkey.

**Keywords:** Pain, Pain Management, Nursing, Bibliometrics, Scientometrics, Nursing Research

## Abstract

**Objective:**

to analyse research articles on pain and nursing issues using bibliometric and scientometric methodologies.

**Method:**

articles in the Web of Science database containing *pain and nurse* and *pain and nursing* were analyzed using scientometric methods through data visualization techniques and advanced text analytics.

**Result:**

among the 107,559 research articles found in the field of nursing, 3,976 of them were written based on the keywords *pain* and *nursing*, and were considered in conformity with the scope of this study. Preliminary analyses indicated that the publications have increased through the years with minor fluctuations. Titles, keywords, and abstracts were analyzed through text analytics to reveal keyword clusters and topic structures. Studies on oncology and pain in the field of nursing have a relatively higher frequency.

**Conclusion:**

the results of the analyses revealed the characteristics of the current literature in a broad range of areas by considering the particular dimensions. Therefore, the findings may support present and future research in this field by shedding light on the networks, trends, and contents in the related literature.

## Introduction

Nurses are the largest group of healthcare professionals providing continuity of care, both in acute and community settings^1^. The basis of nursing care is to ensure that the patient feels comfortable^2^. Pain and the related problems that adversely affect the comfort of patients are among the most common problems faced by nurses during patient care. The International Association for the Study of Pain defines pain as “an unpleasant sensory and emotional experience associated with actual or potential tissue damage, or described in terms of such damage”^3^. Pain is one of the symptoms that should be assessed and managed with a high priority, and nurses are the health professionals who play a primary role in this issue^4^ ^-^ ^6^. Research on pain management and attitudes in the field of nursing has been conducted since 1987^7^. The concept of pain is a subject that also interacts with the subfields of nursing care, such as pain in cancer patients, post-stroke pain, pain in intensive care patients, pain in children, and post-operative pain^8^. 

Bibliometric studies about pain have been conducted under the title of *pain studies* in Africa^9^; *pain research* in Croatia^10^; and *medical and biological pain research* *literature* in the European Union^11^. These studies were assessed in a single country and in a group of countries, but providing limited information when compared to the potential findings of bibliometrics and scientometrics. The study found that 39.86% of studies on pain in children, conducted between 1975 and 2010^12^, were about types of pain, 37.49% were about pain applications, and 25% were about pain evaluations. Additionally, the most cited articles about pain^13^ ^)^ have been analyzed using bibliometric methods on types of pain, such as acupuncture studies^14^, orofacial pain research production^15^ ^)^ and migraine research studies^16^. 

The published research on pain in the related literature has increased, not only in the field of medicine, but also in the field of nursing. The general demographics and emergence of the hidden patterns over the years in the related literature can be extracted through scientometric and bibliometric approaches, with the help of their detailed and analytical techniques. In this context, the studies on pain and pain management can be decomposed into different components concerning the information provided by publishers, e.g., title, author(s), abstract, references, publication information, funding, publication impact factors, location, and keywords. Even though the field of nursing has often been addressed in scientometric or bibliometric studies, pain and pain management have not been investigated from this perspective to date.

Bibliometrics can be considered an essential methodology, used to evaluate the academic performance of nursing studies^17^ ^-^ ^18^. Quantitative bibliometric measures are used to assess the impact of research outputs, and can also be used as tools by librarians to manage collections and provide relevant resources to users^19^ ^-^ ^20^. The bibliometric tools can reveal the trends in nursing terminology and include analyses of core journals, indicators of scholarly output, and the co-author network associated with journal articles^21^. Furthermore, various insights about the intellectual and social structure of a field, as well as research performance and dissemination of ideas can be extracted from the available data with respect to different dimensions, such as authors, documents, journals, words, indicators, metrics, and techniques. That is, counts, correlations, clustering, and network analyses can reveal information about authorship, types of documents cited, journal distributions, and how works are connected by highlighting the patterns, trends, identified interests and spreading^22^ ^-^ ^23^. 

The necessary datasets can be retrieved from numerous online databases, such as Web of Science (WoS) or Scopus^24^. Network analyses and text analytics techniques in the scope of scientometrics are especially useful as a way of mapping a research field, and although they have been widely used in many fields, they have not yet been used in the field of pain and nursing. This motivation determined the goal of this study, which is the investigation of research articles on pain and nursing issues using bibliometric and scientometric methodologies. This study presents the covered topics, trends in the cited journals and authors, the funding in countries, and the status of organizations and works, and thus illuminates the development in this field, providing a broader perspective on the current status of the literature.

## Method

This descriptive and exploratory study can be classified as both bibliometric and scientometric research, since it includes tables and graphs to present descriptive statistics and uses advanced text analytics and network analyses to reveal hidden patterns in the content of abstracts, with regard to the relationships among the terms. The methodology was designed as an end-to-end process, beginning with the dataset retrieval and ending with obtaining the findings from various analyses and tools.

The related data to perform the analyses were extracted from the WoS online platform. The article search was conducted in August 2017, within the core collection of the WoS, on the title, abstract, and keywords of all articles published between January 1, 1975 and July 31, 2017. The retrieved dataset was taken into consideration in the scope of the study as well. The following terms were used in the search strategy: (pain* and nurse*) or (pain* and nursing*). An asterisk was used as a wildcard to retrieve documents containing the words *nurse or nurses,* to deal with the *article* as document type, and *all years* as timespan in the nursing category of the WoS. The resulting record content included the full records and cited references in a plain text and a tab-delimited (for Windows) text file format. 

The data exported in a plain text format was stored in a relational database using the Oracle platform through a novel program developed in the Hypertext Preprocessor Programming (PHP) language, with the aim of obtaining queries with the Structured Query Language (SQL) and performing customized analyses. For the analytical stages of the methodology, several software tools were utilized, i.e., the VOSviewer^25^ and Microsoft Excel for descriptive statistics, network/density visualizations, and clustering on the networks; RapidMiner^26^ for text preprocessing; and a java application for Hierarchical Latent Tree Analysis (HLTA), for topic modeling in the abstracts. 

Bibliometric mapping is a quantitative approach that aimed at visualizing various bibliometric aspects of scientific publications, performed in the form of different networks. In this case, the authors induced scientific landscapes, used for content analysis, and bibliometric networks to present co-authorship and co-citation. For this purpose, the VOSviewer, a software package for analyzing and visualizing large bibliographic datasets^25^, was preferred for the graphical representations of this study concerning various dimensions, such as journals, authors, countries, organizations, and individual publications. The network representations can be built by co-authorship, co-citation, or other bibliographic relationships.

For the text analytics on titles, keywords, and abstracts in the dataset, a text preprocessing data model was performed for tokenization, filtering stop words, and part-of-speech-tags, including names and verbs, stemming, and other filters required in the further analyses. The data model was built using the RapidMiner 7.6 software platform. The data model produced preprocessed text data that were stored in a spreadsheet in which the text for each article was saved in a single cell. To provide the proper inputs for HLTA implementation to obtain the topic structure in the data, the text data in each of the cells of the spreadsheet was converted into a single text file using the Visual Basic for Applications (VBA) as coding tool. The group of text files was then processed through a progressive expectation maximization algorithm for topic detection using the suggested parameters^27^. HLTA provided insights from the content of the articles and shed light on the main topics that appeared in the pain studies that were within the scope of nursing research.

## Results

During this study, 189,885 publications in the nursing category were reached through the WoS databases. This number indicated that the nursing field has a particular place in health research. When this comprehensive dataset was filtered, it was found that 107,559 (56.64%) documents were research articles, and of these articles, 3,976 (3.55%) contained the words nursing* and pain* or nurse* and pain*, in the scope of their topics. This ratio, which is related to the concepts of pain and pain management, constitutes 3.69% of the articles that exist in the field of nursing. Bibliometric performance measures also indicated the position of this research area in the scientific literature^5^, such as *H-index*: 67; *average citations per item*: 10.94; *the sum of times cited*: 43,501 (without self-citations: 37,325), and *the number of citing articles*: 30,324 (without self-citations: 28,209). 

**Figure 1:**
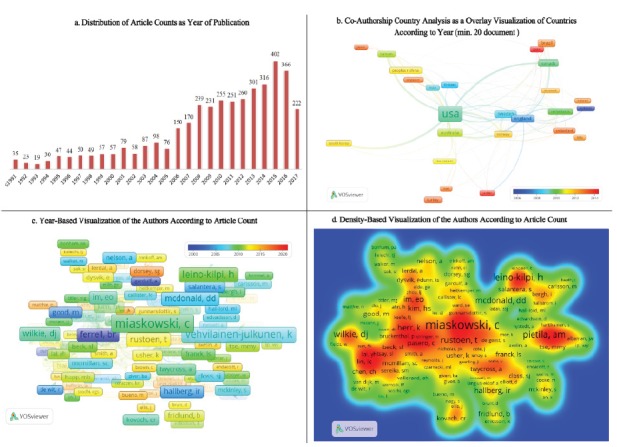
Distribution of article counts, co-authorship country analysis, and year and density-based visualization of the authors. Izmir, Turkey, 2017

The studies overlapping with the words *nursing* and *pain* in the nursing category of the WoS showed an increasing trend over the years. The first high incremental phase was seen in 2006, and the counts have continued to increase. There were fewer articles written in this field in 2017 (Figure 1a). However, this situation occurred because of the time range of the dataset, which was completed in August 2017, prior to the end of the year. Figure 1b details the dimensions of the country according to the distribution by year, and provides co-authorship information. The color legend in Figure 1b defines the time in years, and the font sizes indicate the density of the work in the corresponding country. When the co-authorships in the first five countries, in terms of number of publications, were examined, the relationships were determined as: United States of America (USA)?Canada, South Korea, Taiwan, Australia?England, USA, Singapore, England?Australia, Canada, Netherlands, Sweden?Norway, USA, Australia, and Brazil à USA, Canada, Spain. 

Analyses indicated that 3,976 articles were written by 10,412 different authors. The five most productive authors were: Miaskowski (Number of articles *(n)* = 34, Number of Citations (*C*) = 834); Vehvilainen-Julkunen (*n* = 26, *C* = 362); Leino-Kilpi (*n* = 21, *C* = 172); Pietila (*n* = 21, *C* = 274); and Wilkie (*n* = 21, *C*= 190), respectively. The year-based and density-based visualization of the authors, according to article count, is shown in Figure 1a. In the year-based visualization of the authors, Figure 1c shows a similar structure and legend to that of Figure 1b, where the colors describe the years in which the authors published most of the articles, and font sizes are directly proportional to the number of articles the authors published. The density-based visualization of the authors, presented in Figure 1d, had a structure similar to heat maps, where blue tones showed the authors with the lesser numbers of articles, and red tones showed the authors with the highest number of articles in the related subject.

Different citation metrics may reveal different authors in the top five, because some authors may have a few pioneering studies outstanding in the field. In this context, the first five authors are: Froelicher (*n* = 1, *C*= 360, Average (*Avg*) = 360); Janson (*n* = 1, *C* = 360, *Avg* = 360); Rankin (*n* = 1, *C* = 360, *Avg* = 360); Tanner (*n* = 1, *C* = 345, *Avg* = 345); and Maneesriwongul (*n*= 1, *C* = 316, *Avg* = 316).

When the references that the researchers cited in their studies were analyzed, it was observed that 62,660 references were used in total for 3,976 articles. The first 10 authors referenced by the researchers (authors cited as the first author only) were: McCaffery (*n* = 550), Ferrell (*n* = 411); Melzack (*n* = 373); Herr (*n* = 249); Polit (*n* = 232); Puntillo (*n* = 205); Cleeland (*n* = 196); Gelinas (*n* = 189); Pasero (*n* = 188); and Benner (*n*= 167). Table 1 shows the top-10 most cited articles about the concept of pain in nursing by author, number of citations, year, and journal information.

**Table 1:** The top 10 most cited articles in the related subject. Izmir, Turkey, 2017

Rank	Title	Journal	FYIF^*^	Year	Authors	Authors Count	C^†^
1	Advancing the science of symptom management	Journal of Advanced Nursing	2.612	2001	Dodd, M; Janson, S; Facione, N; Faucett, J; Froelicher, ES; Humphreys, J; Lee, K; Miaskowski, C; Puntillo, K; Rankin, S; Taylor, D	11	365
2	Thinking like a nurse: A research-based model of clinical judgment in nursing	Journal of Nursing Education	1.444	2006	Tanner, CA	1	351
3	Instrument translation process: a methods review	Journal of Advanced Nursing	2.612	2004	Maneesriwongul, W; Dixon, JK	2	320
4	Pain assessment in the nonverbal patient: Position statement with clinical practice	Pain Management Nursing	1.689	2006	Herr, K; Coyne, PJ; Key, T; Manworren, R; McCaffery, M; Merkel, S; Pelosi-Kelly, J; Wild, L	8	217
5	Towards clarification of the meaning of spirituality	Journal of Advanced Nursing	2.612	2002	Tanyi, RA	1	188
6	Factors related to childbirth satisfaction	Journal of Advanced Nursing	2.612	2004	Goodman, P; Mackey, MC; Tavakoli, AS	3	184
7	Chronic illness self-management: Taking action to create order	Journal of Clinical Nursing	1.825	2004	Kralik, D; Koch, T; Price, K; Howard, N	4	180
8	Development and Preliminary Validation of the Pain Assessment Checklist for Seniors With Limited Ability to Communicate	Pain Management Nursing	1.689	2004	Fuchs-Lacelle, Shannon; Hadjistavropoulos, Thomas	2	150
9	Development and evaluation of a multifaceted ergonomics program to prevent injuries associated with patient handling tasks	International Journal of Nursing Studies	4.278	2006	Nelson, A; Matz, M; Chen, FF; Siddharthan, K; Lloyd, J; Fragala, G	6	137
10	Work-related back pain in nurses	Journal of Advanced Nursing	2.612	1996	Hignett, S	1	134

*FYIF: Five-Year Impact Factor; †C: Number of Citations

The first 20 journals in which the authors published their articles about pain are shown in Table 2. In the journal list, the three first, in terms of the number of articles about pain, were: Pain Management Nursing (*N* = 460, *X* = 457, 99.34%); Oncology Nursing Forum (*N* = 859, X = 106, 12.33%); and Journal of Hospice Palliative Nursing (*N* = 472, X = 49, 10.38%). The 20 first journals having the highest number of publications were classified by countries as: USA (*n* = 13), England (*n* = 4), Brazil (*n* = 2), and Scotland (*n* = 1). 

**Table 2:** Top 20 most published journals in a relevant subject. Izmir, Turkey, 2017

Rank	Journal	Publisher Country	Research Domain	FYIF^*^	N^†^	X ^‡^	%^§^	C**
1	Pain Management Nursing	USA	Nursing	1.689	460	457	11.49	3618
2	Journal of Advanced Nursing	USA	Nursing	2.612	7494	416	10.46	9344
3	Journal of Clinical Nursing	England	Nursing	1.825	4438	378	9.50	4952
4	International Journal of Nursing Studies	England	Nursing	4.278	2552	143	3.59	2317
5	Cancer Nursing	USA	Oncology, Nursing	2.193	1827	137	3.44	2910
6	Oncology Nursing Forum	USA	Oncology, Nursing	2.879	859	106	2.66	2026
7	Scandinavian Journal of Caring Sciences	England	Nursing	1.794	1463	62	1.55	906
8	Revista Latino Americana De Enfermagem	Brazil	Nursing	0.884	1513	61	1.53	195
9	Revista Da Escola De Enfermagem Da Usp	Brazil	Nursing	0.648	1589	59	1.48	158
10	Clinical Journal of Oncology Nursing	USA	Oncology, Nursing	0.904	840	52	1.30	520
11	Nursing Clinics of North America	USA	Nursing	0.781	2170	52	1.30	358
12	Journal of Midwifery Women’s Health	USA	Nursing	1.451	994	51	1.28	731
13	Journal of Hospice Palliative Nursing	USA	Nursing	0.759	472	49	1.23	84
14	European Journal of Oncology Nursing	England	Oncology, Nursing	2.155	816	48	1.20	355
15	Orthopaedic Nursing	USA	Nursing, Orthopedics	0.634	467	48	1.20	277
16	Applied Nursing Research	USA	Nursing	1.738	1022	46	1.15	274
17	International Journal of Nursing Practice	USA	Nursing	1.139	834	46	1.15	437
18	Journal of Emergency Nursing	USA	Emergency Medicine, Nursing	1.222	937	46	1.15	276
19	Nursing Research	USA	Nursing	2.113	2133	44	1.10	775
20	Nurse Education Today	Scotland	Education & Educational Research, Nursing	2.636	2719	39	0.98	364

*FYIF: Five-Year Impact Factor; †N: All Published Articles; ‡X: Number of Articles about Pain; §%: Percentage; **C: Number of Citations

In total, 24,795 different sources have been cited in the references of the related research articles. Considering the journals that published the articles, the 20 most cited were: Journal of Advanced Nursing (*n* = 4,372); Pain (*n* = 3,554); Journal of Pain and Symptom Management (*n* = 2,642); Journal of Clinical Nursing (*n* = 1,909); Pain Management Nursing (*n* = 1,488); Oncology Nursing Forum (*n* = 1,356); Cancer Nursing (*n* = 1,243); Journal of the American Geriatrics Society (*n* = 1,224); Nursing Research (*n* = 1,206); International Journal of Nursing Studies (*n* = 1,174); The Latest Medical Research, Reviews, and Guidelines (*n* = 1,083); The Clinical Journal of Pain (*n* = 978); the Journal of Pediatrics (*n* = 831); the British Medical Journal (*n* = 788); Research in Nursing & Health (*n* = 782); Anesthesia & Analgesia (*n* = 719); and The Cochrane Database of Systematic Reviews (*n* = 697). 

Institutions play a critical role for researchers by supporting them in many ways, because the performance of the researchers is an important component of the performance of the institution. The analyses conducted in this regard indicated that 3,976 articles on pain were produced by 3,311 different organizations, and authors from different organizations carried out some of the studies. The most productive institutions in this area were: University of Sao Paulo (Brazil, *n* = 85, 2.13%, *C*= 239); University of California San Francisco (USA, *n* = 77, 1.20%, *C* = 1,510); University of Pennsylvania (USA, *n*= 52, 1.30%, *C* = 728); Karolinska Institute (Sweden, *n*= 48, 1.20%, *C* = 494); University of Wisconsin (USA, *n*= 45, 1.13%, C = 907); University of Iowa (USA, *n*= 44, 1.10%, *C* = 803); University of Washington (USA, *n*= 42, 1.05%, *C* = 720); Hong Kong Polytech University (Hong Kong, *n* = 41, 1.03%, *C* = 706); University of North Carolina (USA, *n* = 39, 0.98%, *C* = 381); and University of Oslo (Norway, *n*= 35, 0.88%, *C* = 327).

The top 10 countries that produced articles about pain and nursing were: USA (*n* = 1,674; *C* = 19,307); Australia (*n* = 272; *C* = 3,156); England (*n* = 265; *C*= 3,608); Sweden (*n* = 244; *C* = 3,293); Brazil (*n*= 222; *C* = 653); Canada (*n* = 206; *C* = 3,165); Turkey (*n* = 155, *C*= 978); Taiwan (*n* = 129; *C*= 1,592); China (*n* = 117; C = 1,429); and South Korea (*n* = 109, *C* = 674). 

Bibliometrics is an important tool for measuring academic and organizational performance. The quantity and quality of research produced by individual researchers, research groups, and universities, are important measurements of their success and contribution to the productivity of the economy^24^. The top three universities that contributed the most to this field in the world ranking were: University of Sao Paulo (Brazil); University of California San Francisco (USA); and University of Pennsylvania (USA). The USA was the most remarkable, and ranked first in the number of universities, journals, and articles. Brazil was ranked in the first five countries with regard to the number of publications, and was ranked first in the universities. In recent years, there has been an increase in the number of publications about the related subject in countries such as Iran, Turkey, and Spain.

Every paper in the WoS Core Collection was assigned to at least one of the subject categories according to the published source, and this information was stored in the field WoS Categories of the corresponding record. The fields of the 3,976 articles investigated in this study were retrieved from the nursing category, and the articles that were also indicated in the other fields were: oncology (*n*= 369, 9.28%); pediatrics (*n*= 85, 2.13%); obstetrics-gynecology (*n* = 69, 1.73%); geriatrics gerontology (*n*= 67, 1.68%); and gerontology (*n* = 67, 1.68%).

**Figure 2:**
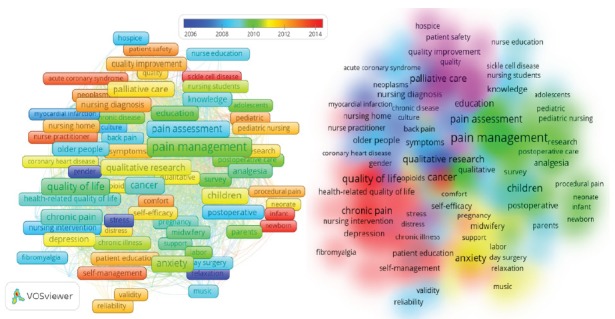
Co-occurrence author keywords analysis as an overlay visualization and cluster density. Izmir, Turkey, 2017

Keywords were also analyzed to map the distribution of the articles containing these words. The authors of the articles defined 5,745 keywords in total; 194 of these words were repeated 10 times or more. The articles were subjected to a cluster analysis concerning their keywords, and eight clusters were obtained, as depicted in Figure 2.

The 20 first most commonly used keywords in the articles were: pain management (*n* = 207); quality of life (*n* = 113); cancer (*n* = 96); pain assessment (*n*= 92); nursing care (*n* = 92); children (*n* = 85); anxiety (*n*= 80); palliative care (*n* = 74); postoperative pain (*n*= 70); chronic pain (*n* = 70); qualitative research (*n* = 69); education (*n* = 47); evidence-based practice (*n* = 46); depression (*n* = 44); emergency department (*n* = 43); dementia (*n* = 42); assessment (*n* = 41); knowledge (*n* = 40); occupational health (*n* = 38); and older people (*n* = 37). 

**Figure 3:**
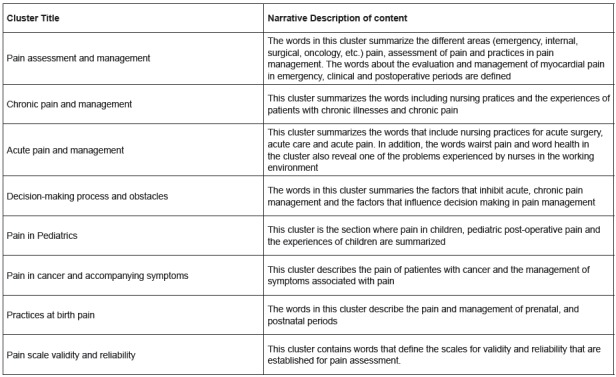
Cluster title and description of author keywords. Izmir, Turkey, 2017

The zoom in-zoom out feature of the VOSviewer program provided a detailed analysis of word phrases. The related articles were analyzed with regard to the occurrences of word phrases in the VOSviewer (including at least 10 occurrences), and eight clusters were obtained from author keywords. Clusters related to the word phrases were also analyzed in detail using an Oracle database and SQL. Furthermore, HLTA was performed to extract the detailed topic structure, considering not only the keywords, but also the title and abstract of each article. Finally, by considering the results obtained from the VOSviewer and HLTA, the clusters were titled and were interpreted, as presented in Figure 3. 

The HLTA results also highlighted some important issues about the details of the contents of the related articles. For example, the most commonly studied topics were: visual analogue scale in pain assessment; students’ knowledge and skill in assessing the pain of a patient; pain assessment scale validity and reliability of different patients; qualitative studies in pain; pharmacological treatment of pain; experimental control studies in pain; pain prevalence, symptoms, and pain in oncologic patients; and pain in children and newborns. 

## Discussion

Research articles about pain constituted 3.55% of all nursing publications, and this percentage indicated that the subject, *pain* *in nursing,* is an intensively studied subject in the related field, and had an increasing trend during the period from 1975 to 2017. This trend can be explained by the fact that pain is a problem that many patients experience and is subject to various research attempts in the evaluation and management of pain^4^. The H-index value of the topic is 67, and high enough to support these observations. Thus, the past and recent trends can provide much useful information to researchers.

In this study, 9.28% of pain-related articles in the field of nursing were related to the field of oncology. This result supports the findings that the first three words used by the authors are: cancer (*n* = 96); that one of the eight clusters is pain in cancer and accompanying symptoms; the headline symptoms and pain in cancer were determined in HLTA. These results revealed that studies of pain in the nursing field are associated with pain, pain assessment, and pain management in cancer patients. Pain is the most common symptom that has been experienced and feared the most by cancer patients^28^, accordingly, the words *quality of life* and *stress* are intensified in the keyword distribution. The results of this study highlight the effect of stress for both patients and work health. Pain is a problem that negatively affects the lives of working people^14^. *Waist pain and stress* in employees are the keywords in the *acute pain and management* cluster. Furthermore, HLTA analysis showed the intensity of the articles about scale validity and reliability for pain assessment in the study. 

The studies about the establishment of scales for different patient groups and the reliability of validity were observed as an increasing issue. When the distribution of keywords according to years was examined, up to the year 2008*, pain management*, and *pain and child* studies had a relatively higher rank in the field of pain management, whereas *dementia*, *palliative* *care*, and *pain* *in* *cancer* studies were identified as more popular subjects after 2008, supporting the study^29^. 

A major issue for the contemporary scholar is to disseminate information in an increasingly competitive market. While nursing researchers have many options regarding where to publish, choosing a publication venue is rarely a clear-cut decision^30^. In light of the findings of the analyses, researchers and practioners studying pain and pain management in nursing care can find many useful insights and information about the current status of the literature and the recent trends, which may support their research in the present and future.

## Conclusion

This study focused on revealing the current status of the literature about pain and pain management in the field of nursing with respect to the particular dimensions, such as the distribution of the authors, journals, institutions, countries, keywords in terms of years, citations, networks, topic detection, and document clustering over keyword distributions. The study revealed that articles about pain have primarily focused on children, elderly, and oncologic patients in recent years. It was determined that pain studies should not only be focused on patients, but also on pain situations related to the working conditions of health professionals. It is suggested that it would be beneficial to investigate the content of pain studies in specific areas in more detail, such as oncology or palliative care patients in certain age groups, i.e., the elderly or children, using scientometrics.

This study revealed the general pattern of pain studies in the nursing literature. In light of the information provided by the authors, researchers working on pain and pain management can follow the publications and journals that have made significant contributions to the field to improve the quality of their research and plan appropriate future work according to the trends provided in the tables, networks, and the content pattern of the articles. The use of the techniques related to topic modeling provides insights about the content of the publications. In this study, topic modeling using HLTA provided significant information to strengthen the visualization of keyword densities and the network. Hence, topic modeling may be considered as a constant component of scientometric tools and studies.
